# Iodine status of euthyroid adults: A cross‐sectional, multicenter study

**DOI:** 10.1002/jcla.22837

**Published:** 2019-02-08

**Authors:** Danchen Wang, Songlin Yu, Yicong Yin, Shaowei Xie, Qian Cheng, Honglei Li, Xinqi Cheng, Ling Qiu

**Affiliations:** ^1^ Department of Clinical Laboratory, Peking Union Medical College Hospital Peking Union Medical College & Chinese Academy of Medical Science Beijing China

**Keywords:** iodine, nutrient, thyroid disease, thyroid function, thyroid‐stimulating hormone, urine

## Abstract

**Background:**

Iodine, an essential nutrient, is the most important trace element in thyroid hormone synthesis and maintenance of thyroid function. This study investigated the iodine nutrition status in healthy Chinese adults and assessed the relationship between urinary iodine concentration (UIC) and thyroid hormone levels.

**Methods:**

A cross‐sectional, multicenter study was conducted between October 2017 and January 2018, with 1017 adults recruited from five cities in China. All subjects underwent thyroid ultrasonography, and only those with normal results were included in the study. UICs were measured by inductively coupled plasma mass spectroscopy and adjusted using urine creatinine levels. Thyroid hormone levels were measured using an automated immunoassay analyzer.

**Results:**

The median UIC and adjusted UIC were 134.0 µg/L and 114.2 µg/g, respectively. UIC was not significantly different between males and females (*P* = 0.737). However, the adjusted UIC was significantly different between sexes (*P* < 0.001). The median UIC was higher than 100 µg/L. According to the World Health Organization criterion (100 µg/L), the total prevalence of iodine deficiency is 33.1% (n = 271). The prevalence rates of iodine deficiency in our study were 33.2% and 32.9% in males and females, respectively, and had no difference between sexes and among cities (*P* > 0.05). Serum thyroid‐stimulating hormone (TSH) levels increased when UIC increased. The Kruskal‐Wallis test showed no significant differences in free triiodothyronine, free thyroxine, and TSH, with different levels of UIC (all *P* > 0.05).

**Conclusions:**

Chinese adults with normal thyroid structure have relatively sufficient iodine levels.

## 1 INTRODUCTION

Iodine is an essential nutrient and the most important trace element in the synthesis of thyroid hormones and maintenance of normal thyroid function.^1^ The thyroid gland plays important roles in regulating metabolism and promoting normal development of cardiovascular, reproductive, and nervous systems.^2^ Thyroid hormones are reliable indicators of thyroid function, especially thyroid‐stimulating hormone (TSH) and free thyroxine (FT4). Iodine nutrition is a key determinant of thyroid disease risk, and continued vigilance against iodine deficiency remains essential in developed and developing countries.^3^ Deficiency or excessive iodine intake is related to hypothyroidism or goiter formation.^4^, ^5^ While short‐term lack of iodine can be compensated by the body, abnormal long‐term iodine intake can lead to thyroid hormone synthesis disorders, thyroid dysfunction, or thyroid disease.^5^

Iodine deficiencies were prevalent in China until the introduction of universal salt iodization (USI) in 1995.^6^, ^7^ In 2000 and 2011, the USI standards were adjusted, and the principle of individualization of cities was proposed.^8^ Iodine status has been historically assessed by goiter rates,^9^ although the definition of goiter is subjective. Currently, the iodine status is usually evaluated using the urine iodine concentration (UIC), which, based on population studies, is a recommended and reliable method to assess iodine status.^9^, ^10^ Spot urine is a cost‐efficient, easily obtainable, and well‐acceptable method, since >90% of dietary iodine appears in urine within 24‐48 hours.^11^

Many studies have focused on the evaluation of iodine status and its association with thyroid disease using UIC. These studies reported that the iodine status in China was sufficient or adequate and that excessive iodine intake led to increased prevalence of thyroid disease.^3^, ^8^, ^12^, ^13^ However, there is a lack of studies investigating iodine status in apparently healthy volunteers with normal thyroid ultrasonography tests.

Therefore, our study examined the iodine nutrition status in adults whose thyroid structure was normal, and the association between UIC and thyroid hormones, to provide reasonable suggestions to the government under the implementation of USI.

## 2 MATERIALS AND METHODS

### 2.1 Study population

Analytical data used in this study were collected from five cities (Beijing, Guizhou, Xinjiang, Shandong, and Heilongjiang) during October 2017 to January 2018. Participant inclusion criteria were as follows:

1. Resident of locality for >1 year;
2. Age ≥ 18 years;
3. Normal thyroid ultrasonography measurement.

Participant exclusion criteria were as follows:

1. Had high fever in the past 15 days;
2. Presence of serious systemic disease including cardiovascular, renal, gastrointestinal, pulmonary, or thyroid disease, or cancer;
3. Took thyroid medication in the past 15 days;
4. Was a hospital inpatient or seriously ill during the previous 4 weeks;
5. Surgery in the past 6 months;
6. Female participants, pregnant, breastfeeding, or within 1 year after childbirth;
7. On a high‐iodine diet or consumed seafood including kelp, sea fish, crab, shrimp, and shellfish in the past 3 days.

A total of 1017 apparently healthy participants aged 18‐82 years were enrolled in this study. This study was approved by the Ethics Committee of the Institute of Peking Union Medical College Hospital. All participants studied were informed in writing of the intended use of their samples, and each participant provided written consent.

### 2.2 Data collection and physical examination

Data including demographic characteristics and medical history were collected from a representative sample of the study via a standard questionnaire. Body weight was measured on a calibrated beam scale, and height was measured in triplicate. Body mass index (BMI) was calculated as body weight divided by the square of the height (kg/m^2^). Blood pressure (BP) was measured three times after the participant rested quietly for at least 10 minutes, and the average of three measurements was used. Current smoking status was classified as a self‐reported response of “yes” to the question “Do you smoke now?” We also evaluated the UIC distribution between intake iodine salt and noniodine salt among 693 subjects who self‐reported a response to the question “do you consume iodine salt during breakfast, lunch, or dinner?” All participants underwent thyroid ultrasonography examination performed by trained technicians.

### 2.3 Laboratory measurement

All subjects were advised to have a bland diet before blood testing. Following overnight fasting, blood was drawn from the antecubital vein of the arm. Spot urine samples were also collected. Blood specimens were centrifuged at 3000 rpm/min for 10 minutes. All samples were sent to the laboratory and stored at −80°C until tested.

Calibration and quality controls (Lyphochek^®^ Control) were performed before the analyses to monitor instrument precision. Measurements were performed according to the standard operation procedure. Instrument calibration and preventive maintenance were performed annually. We also participated in External Quality Assessments by the National Center for Clinical Laboratories and College of American Pathologists to guarantee the accuracy and reliability of results.

UIC was measured by inductively coupled plasma mass spectroscopy. Urine creatinine was measured using Beckman AU 2700 Automatic Biochemical Analyzer. Serum lipoprotein, including total cholesterol (TC) and triglycerides (TG), and fasting blood glucose (FBG) were measured. Thyroid hormones including free triiodothyronine (FT3), FT4, and TSH were measured using Beckman DXI 800 chemiluminescent immunoassay. The reference range for FT3, FT4, and TSH were 2.5‐3.9 pg/mL, 0.61‐1.12 ng/dL, and 0.38‐5.33 mIU/L respectively. The precision of FT3, FT4, and TSH measurements was assessed according to the Clinical Laboratory and Standard Institution EP‐15A2 protocol. We previously used this method for measuring UIC. The results revealed that the inter‐run coefficients of variation (CVs) and total CVs for urine iodine were 3.5%‐6.7% and 3.9%‐6.7%, respectively. The intra‐ and interassay coefficient of variation for FT3, FT4, and TSH were 5.4%‐8% and 5.8%‐7.6%, 4.1%‐7.6% and 1.7%‐6.9%, and 2.4%‐3.9% and 2.2%‐3.7%, respectively, which meet the clinical values.

All laboratories participating in the survey followed the same internal quality control program that was standardized by the Peking Union Medical College Hospital.

### 2.4 Iodine status

The iodine status of subjects was assessed by median UIC based on the World Health Organization (WHO) recommendations. According to iodine nutrition epidemiologic criteria of WHO, a population's median UIC of <100, 100‐199, 200‐299, and ≥300 µg/L is each representative of insufficient, adequate, above requirements, and excessive iodine intake. In this study, UIC of enrolled subjects was classified by <100, 100‐299, and ≥300 µg/L. Furthermore, the prevalence of iodine deficiency was defined as proportion of subjects with a UIC <100 µg/L. The UIC values were adjusted using urine creatinine.^9^ Urine iodine can be expressed in a relationship with creatinine excretion (µg iodine/g creatinine) also called adjusted urine iodine concentration equation 1.^9^

(1) $$\begin{array}{c} AdjustedUIC= \end{array}\left(\frac{\mathrm{UIC}}{\mathrm{urinecreatinine}}\right)\times 8.84$$

### 2.5 Statistical analysis

SPSS 20.0 (SPSS Inc., Chicago, IL, USA) and Excel 2016 statistical software (Microsoft Corporation, Redmond, WA, USA) were used for data analysis. Normally distributed data were presented as mean and standard deviation (SD), while skewed data were expressed as median (percentiles). Categorical variables were presented as a number (percentile). Group differences of normally distributed values were compared using the *t* test or one‐way ANOVA, and skewed data were compared using the Mann‐Whitney *U* or Kruskal‐Wallis test. Group differences of categorical variables were compared using the chi‐square test. *P* < 0.05 was defined as statistically significant.

## 3 RESULTS

### 3.1 Characteristics of participants

A total of 1017 adults from five cities were recruited for this study. Subsequently, 198 subjects lacking complete information or urine iodine measurements were excluded. Ultimately, 819 subjects with complete information and UIC and urine creatinine measurements who met the inclusion criteria were used in the final analysis. Baseline characteristics of study subjects are shown in Table 1. Among 819 subjects, the average age, BMI, systolic blood pressure (SBP), and diastolic BP (DBP) were 41.3 ± 13.2 years, 23.3 ± 3.6 kg/m^2^, 122 mm Hg, and 76 mm Hg, respectively. One‐way ANOVA showed that there were significant statistical differences in age, BMI, SBP, DBP, FBG, TG, TC, FT3, FT4, and TSH among different cities (*P* < 0.001).

**Table 1:** Baseline characteristics of subjects

Area	Beijing	Guizhou	Xinjiang	Shandong	Heilongjiang	Total
N	150	192	186	143	148	819
Sex ratio (man) (%)	51.3	53.1	45.7	51.7	39.9	48.5
Age*	36.0 ± 9.7	43.5 ± 15.0	42.3 ± 13.4	41.6 ± 12.6	42.4 ± 12.7	41.3 ± 13.2
BMI (kg/m^2^)*	22.7 ± 2.6	23.1 ± 3.2	23.3 ± 2.9	24.5 ± 5.4	23.1 ± 2.9	23.3 ± 3.6
SBP (mm Hg)*	117 ± 13	122 ± 15	121 ± 12	131 ± 17	122 ± 16	122 ± 15
DBP (mm Hg)*	71 ± 9	74 ± 9	73 ± 8	81 ± 12	83 ± 12	76 ± 11
FBG (mmol/L)*	5.1 ± 0.7	5.2 ± 1.1	4.8 ± 0.7	5.7 ± 1.3	5.0 ± 0.5	5.1 ± 1.0
TG (mmol/L)*	1.12 ± 0.84	1.63 ± 1.03	1.56 ± 1.13	1.37 ± 0.94	1.13 ± 0.67	1.38 ± 0.97
TC (mmol/L)*	4.46 ± 0.80	4.93 ± 0.98	4.53 ± 0.88	5.05 ± 0.96	4.66 ± 0.69	4.73 ± 0.90
FT3 (pg/mL)*	3.42 (3.17, 3.66)	3.44 (3.21, 3.73)	3.21 (3.01, 3.56)	3.54 (3.28, 3.80)	3.21 (2.97, 3.42)	3.36 (3.10, 3.65)
FT4 (ng/dL)*	0.93 (0.86, 1.01)	0.89 (0.82, 0.96)	0.90 (0.82, 0.99)	0.89 (0.81, 0.97)	0.93 (0.85, 1.03)	0.91 (0.83, 0.98)
TSH (mIU/L)*	1.84 (1.38, 2.51)	2.15 (1.56, 3.31)	2.18 (1.59, 3.12)	1.87 (1.36, 2.57)	1.67 (1.07, 2.22)	1.97 (1.40, 2.75)

BMI, body mass index; Cr, creatinine; DBP, diastolic blood pressure; FBG, fasting blood glucose; SBP, systolic blood pressure; TC, total cholesterol; TG, triglyceride.

* *P* < 0.05, indicates statistical significance in different areas.

### 3.2 Iodine status of the population

The median UIC and adjusted UIC (*P*_25_, *P*_75_) were 134.0 (86.6‐201.4) µg/L and 113.5 (73.7‐198.2) µg/g, respectively. The Kolmogorov‐Smirnov‐Wallis test revealed that the distribution of UIC was skewed. The Kruskal‐Wallis test demonstrated that there were no significant differences in the UIC levels between the sexes (*P* = 0.737), but there were differences in the adjusted UIC between the sexes (*P* < 0.001). According to WHO criteria, prevalence of iodine deficiency, adequate iodine, and excessive iodine was 33.1% (n = 271), 57.1% (n = 468), and 9.8% (n = 80), respectively. The prevalence rates of iodine deficiency were 33.2% and 32.9%, in males and females, respectively, while the chi‐square test revealed no difference between sexes. The prevalence of iodine deficiency among Beijing, Guizhou, Xinjiang, Shandong, and Heilongjiang was 34.7%, 32.3%, 37.1%, 31.5%, and 29.1%, respectively, and showed no statistical differences (*P* > 0.05).

### 3.3 UIC according to age and geography

Table 2 shows UIC with age and geography. The median UIC decreased as the age increased (*P* = 0.005), and the 18‐29 age‐group showed the highest median UIC. Additionally, the median UICs for all age‐groups were above that required for iodine. When urine creatinine was used to adjust the UIC, median adjusted UIC decreased while age increased in those younger than 49 years of age. The 50‐59 age‐group showed the statistically highest median adjusted UIC (*P* = 0.027). Heilongjiang had a significantly higher median UIC and adjusted UIC than other cities, although the median UIC among cities did not show statistical difference.

**Table 2:** Iodine status of the study population in China classified by the World Health Organization criteria

	N (%)	UIC (µg/L) Median & percentile	Adjusted UIC (µg/g) Median & percentile	<100 µg/L N (%)	100‐299 µg/L N (%)	≥300 µg/L N (%)
Age‐group (years)						
18‐29	196 (23.9)	151.9 (97.6, 214.5)	112.3 (74.6, 188.6)	55 (28.1)	114 (58.2)	27 (13.8)
30‐39	208 (25.4)	144.0 (90.7, 204.2)	107.2 (63.1, 185.9)	58 (27.9)	130 (62.5)	20 (9.6)
40‐49	191 (23.3)	125.4 (73.3, 202.1)	104.0 (80.3, 164.5)	77 (40.3)	95 (49.7)	19 (10.0)
50‐59	137 (16.7)	126.1 (80.1, 187.5)	140.0 (79.1, 243.5)	48 (35.0)	81 (59.1)	8 (5.8)
≥60	87 (10.6)	116.1 (77.7, 171.4)	129.2 (87.2, 241.5)	33 (37.9)	48 (55.2)	6 (6.9)
Geographic						
Beijing	150 (18.3)	133.8 (72.8, 194.3)	86.8 (54.4, 129.0)	52 (34.7)	86 (57.3)	12 (8.0)
Guizhou	192 (23.4)	129.1 (88.6, 187.7)	131.1 (81.3, 209.8)	62 (32.3)	116 (60.4)	14 (7.3)
Xinjiang	186 (22.7)	127.8 (82.3, 207.1)	110.0 (77.4, 185.6)	69 (37.1)	98 (52.7)	19 (10.2)
Shandong	143 (17.5)	139.7 (89.5, 210.0)	102.6 (67.5, 173.2)	45 (31.5)	80 (56.0)	18 (12.6)
Heilongjiang	148 (18.1)	144.6 (94.2, 218.2)	173.7 (98.2, 333.7)	43 (29.1)	88 (59.5)	17 (11.5)
Total	819 (100)	134.0 (86.6, 201.4)	113.5 (73.7, 198.2)	271 (33.1)	468 (57.1)	80 (9.8)

UIC, urinary iodine concentration.

The prevalence of iodine deficiency of the 40‐49 age‐group was significantly higher than other groups. The prevalence of iodine deficiency showed statistically significant change with age according to UIC (*P* = 0.034). Differences in the prevalence of iodine deficiency among cities exhibited no statistical significance as seen with the chi‐square test (*P* = 0.586).

### 3.4 UIC and thyroid function

The median concentrations of FT3, FT4, and TSH in the subjects were 3.36 pg/mL, 0.91 ng/dL, and 1.95 mIU/L, respectively. Table 3 shows changes in serum FT3, FT4, and TSH concentrations according to UIC categories. The serum TSH level showed an increasing trend as UIC was increasing, but Kruskal‐Wallis test demonstrated that there were no differences in TSH with UIC (all *P* > 0.05). The serum FT4 levels did not show statistically and clinically significant changes with UIC (*P* = 0.108). The interquartile range (IQR) of FT3 and TSH increased with increasing UIC. Conversely, the IQR of FT4 decreased with increasing UIC.

**Table 3:** Thyroid hormone concentrations according to UIC

UIC (µg/L)	N (%)	FT3 (pg/mL)			FT4 (ng/dL)			TSH (mIU/L)		
		Median	*P*_25_, *P*_75_	IQR	Median	*P*_25_, *P*_75_	IQR	Median	*P*_25_, *P*_75_	IQR
<100	271 (33.1)	3.33	3.09, 3.64	0.55	0.91	0.83, 0.99	0.16	1.90	1.36, 2.51	1.15
100‐299	468 (57.1)	3.37	3.11, 3.67	0.56	0.91	0.83, 0.98	0.15	2.01	1.41, 2.75	1.34
≥300	80 (9.8)	3.40	3.04, 3.61	0.57	0.89	0.81, 0.95	0.14	2.22	1.55, 3.13	1.58
*P* for trend		0.769			0.108			0.073		

FT3, free triiodothyronine; FT4, free thyroxine; TSH, thyroid‐stimulating hormone; UIC, urinary iodine concentration.

FT3, FT4, and TSH data are presented as median and percentile.

### 3.5 Relation of UIC and other indicators

Among the enrolled subjects, 712 answered the question “Are you smoking now?” and the median UIC of these subjects was 137.8 (89.0, 206.6) µg/L. Of these 712 participants, 84.3% (n = 600) and 15.7% (n = 112) were nonsmoking and smoking, respectively. Iodine deficiency occurred in 32.0% non smoking subjects and 30.4% smoking subjects. There was no difference in the median UIC between non smoking and smoking subjects (135.2 µg/L vs 142.9 µg/L, *P* = 0.979). The median UIC of subjects who consumed iodine salt was higher than that in subjects who consumed non‐iodine salt (143.9 [88.8, 209.6] µg/L vs 131.2 [89.5, 198.1] µg/L). Furthermore, we investigated the correlation between UIC and other indicators. The correlations between UIC and indicators using Spearman correlation analysis are shown in Table 4. There was a statistically significant negative correlation between UIC and age, where UIC decreased as age increased. The relationship between UIC and adjusted UIC showed a positive correlation (*P* < 0.05).

**Table 4:** The correlation between UIC and indicators using Spearman correlation analysis

	Male	*P*	Female	*P*	Total	*P*
Age	−0.125	0.013	−0.134	0.006	−0.128	<0.001
Height (cm)	−0.030	0.550	0.115	0.018	0.021	0.543
Weight (kg)	−0.040	0.429	0.059	0.223	−0.005	0.895
BMI (kg/m^2^)	−0.020	0.686	−0.017	0.727	−0.018	0.608
SBP (mm Hg)	−0.050	0.322	−0.032	0.512	−0.050	0.152
DBP (mm Hg)	−0.017	0.741	0.001	0.976	−0.011	0.745
FBG (mmol/L)	−0.028	0.578	−0.065	0.184	−0.048	0.171
TC (mmol/L)	−0.035	0.492	−0.049	0.313	−0.044	0.215
TG (mmol/L)	0.016	0.754	−0.060	0.219	−0.027	0.436
HDL‐C (mmol/L)	−0.002	0.975	0.032	0.529	0.018	0.609
LDL‐C (mmol/L)	−0.035	0.503	−0.066	0.190	−0.055	0.126
FT3 (pg/mL)	0.049	0.328	0.003	0.951	0.021	0.541
FT4 (ng/dL)	−0.061	0.223	−0.015	0.763	−0.041	0.246
TSH (mIU/L)	0.121	0.016	−0.010	0.830	0.051	0.143
Urine creatinine (mg/dL)	0.289	<0.001	0.339	<0.001	0.311	<0.001
Adjusted creatinine (μg/g)	0.489	<0.001	0.472	<0.001	0.469	<0.001

BMI, body mass index; Cr, creatinine; DBP, diastolic blood pressure; FBG, fasting blood glucose; FT3, free triiodothyronine; FT4, free thyroxine; HDL‐C, high‐density lipoprotein‐cholesterol; LDL‐C, low‐density lipoprotein‐cholesterol; SBP, systolic blood pressure; TC, total cholesterol; TG, triglyceride; TSH, thyroid‐stimulating hormone.

## 4 DISCUSSION

This cross‐sectional study includes the latest survey to date examining the iodine status, and the association between UIC and thyroid hormones, in adults with a normal thyroid ultrasound in China. Few studies have focused on the iodine status of a population with normal thyroid structure. We found that the median UIC was 134.0 µg/L, demonstrating that the iodine levels in Chinese adults were sufficient according to the WHO criteria. Notably, the prevalence of iodine status with a median UIC <100 µg/L was 33.1%.

Because UIC is an indicator to estimate a population's iodine status, it might not be suitable for determining a subject's iodine status.^9^ To ensure the appropriate evaluation of iodine status, we simultaneously measured the creatinine level with spot urine samples, which was used to adjust UIC. However, most studies used only UIC to estimate the iodine status of a population.^3^, ^14^ This study demonstrated that the median UIC varied with age but not with geographic location. A recent study with subjects aged 20 years and older also reported that the median UIC decreased according to age, supporting our data.^12^ We also found that the median adjusted UIC varied by age and geographic location although we did not find various regularities between UIC and adjusted UIC.

The iodine nutrition status of the Chinese population has been suggested to be sufficient in several studies.^3^, ^14^, ^15^ Shan et al reported a median UIC of 205 µg/L in 15 008 healthy adults from 10 cities in eastern and central China.^14^ Another study reported the median UIC of a population without thyroid nodules as 143.1 µg/L.^3^ The iodine nutritional status in the adult population of the Shandong province was reported to have a median UIC of 248.5 µg/L.^15^ In this study, the Chinese population had above sufficient levels of iodine. The UIC in this study was lower than in the previous study. It might be that the UIC was affected by many factors, such as the place of residence (inland, seashore), eating habits, and economic development. However, the overall UIC showed the iodine levels to be sufficient in China.

In this study, the subgroups with a higher UIC were associated with a higher median serum TSH, but not with statistical significance, and a relationship between UIC and FT3 or FT4 levels was not evident. Due to the large interindividual variation in the ability of the thyroid to adapt, thyroid hormones, including FT3, FT4, and TSH, are not considered sensitive indicators of the population iodine status.^10^ Evidence suggests that levels of thyroid hormones will remain within normal range in mild iodine deficiency, while the hormone levels will fall outside the normal ranges only in cases of severe iodine deficiency.^1^ Several studies reported the relationship between UIC and thyroid functions.^5^, ^14^, ^16^ A Korean study reported that the serum TSH and FT4 levels showed statistically significant as UIC.^12^ The prevalence of clinical hypothyroidism, subclinical hypothyroidism, and positive thyroid antibodies, assessed with UIC, was significantly higher in individuals with more than adequate iodine intake, than in individuals with adequate iodine intake.^14^ Although no statistical significance was observed between UIC and thyroid hormones in our study, it is important to control the iodine nutrition intake.

This study demonstrated that the median UIC in smokers was higher than in nonsmokers, but not with statistical significance. Kang et al reported that active smokers had significantly lower iodine levels than passive smokers and nonsmokers.^17^ Regardless of smoking status, both groups were associated with decreasing serum TSH levels, which might be related to lower urinary iodine levels.^17^ It was unclear whether smoking decreased the urinary iodine levels until now.

A strength of this study is that it is the latest study to report an association between UIC and its relationship with thyroid hormones in a Chinese population whose thyroid ultrasonography tests were normal. Additionally, we used urine creatinine adjusted UIC to evaluate the iodine status to ensure the appropriate evaluation of iodine nutritional status. This study still has several limitations. An important limitation is the lack of information on iodine intake via medications, or other sources. Lastly, we used UIC to assess the iodine status of a population. Spot urine sample UIC has been well documented as a suitable indicator for assessment of a population's iodine status. Therefore, currently it is the most suitable indicator to assess iodine status in a population‐based study.

In conclusion, the iodine status of apparently healthy Chinese adults was found to be sufficient. However, salt iodization is still necessary to prevent iodine deficiency.

## CONFLICT OF INTEREST

The authors have no conflict of interests.

## AUTHOR CONTRIBUTIONS

DCW, SLY, HLL, SWX, QC, and LQ performed the experiments. DCW, SLY, YCY, and XQC analyzed the data. DCW, SLY, and HLL wrote the article. DCW, HLL, LQ, and YCY revised the article. DW and SY contributed equally to this article. All the authors have accepted responsibility for the entire contents of this article and approved its submission*.*
